# Hypoxia-mimetic by CoCl2 increases SLC7A5 expression in breast cancer cells in vitro

**DOI:** 10.1186/s13104-023-06650-2

**Published:** 2023-12-11

**Authors:** Leonora Canhasi, Elisabet Tina, Anna Göthlin Eremo

**Affiliations:** 1 School of Medical Sciences, Faculty of Medicine and Health, Örebro University, Örebro, 701 82 SE Sweden; 2 Department of Clinical Research Laboratory, Faculty of Medicine and Health, Örebro University, Örebro, 701 82 SE Sweden

**Keywords:** Endocrine Breast cancer, Hypoxia, LAT1, SLC7A5, CoCl_2_

## Abstract

**Objective:**

Increased expression of the amino acid transporter solute Carrier Family 7 Member 5 (SLC7A5) has been observed in neoplastic cells during hypoxic conditions in vitro, indicating an adaptation for cell survival. To further explore this, we evaluated hypoxia-mimetic by CoCl2 as a model for hypoxia in breast cancer cell lines and the effect on SLC257A5 expression. Four different breast cancer cell lines (MCF7, T-47D, BT-474 and ZR-75-1) were exposed to 100 µM CoCl_2_ for 48 h. Subsequently, cell viability, gene- and protein expression analyses were performed.

**Results:**

The gene expression of VEGF, a marker of hypoxia, was significantly elevated in all four cell lines compared to the control (p < 0.0001), indicating that CoCl_2_ exposure generates a hypoxic response. Moreover, CoCl_2_ exposure significantly upregulated SLC7A5 gene expression in T-47D (p < 0.001), BT-474 (p < 0.0001) and ZR-75-1 (p < 0.0001) cells, as compared to vehicle control. Immunofluorescence staining showed increased SLC7A5 protein expression in MCF7, T-47D and BT-474 cells compared to vehicle control. This report suggests that hypoxia-mimetic by CoCl_2_ can be used as a simple model for inducing hypoxia in breast cancer cell lines and in fact influence SLC7A5 gene and protein expression in vitro.

## Background

An increasing number of studies have associated the transmembrane amino acid transporter SLC7A5, also known as large neutral amino acid-transporter 1 (LAT1), with worse outcome in cancer [1]. As SLC7A5 relates to unfavorable prognostic factors and chemoresistance, the protein may be a potential target for treatment with inhibitors [2]. SLC7A5 plays a crucial role in transporting amino acids, such as leucine, tyrosine and tryptophan, which are required for sustaining biological functions. Upregulation of SLC7A5 provides cancer cells with advantages including increased access to necessary building blocks for protein synthesis. Additionally, SLC7A5 activates the nutrient signaling pathway Mechanistic Target of Rapamycin Kinase Complex 1 (mTORC1) leading to changes in cellular metabolism [3]. The expression of SLC7A5 has been shown to be regulated by the oncogenic c-Myc in pancreatic cancer cells in vitro [4], and may be altered as a result from adaption to hypoxic microenvironment. In a previous study, we found that SLC7A5 is positively correlated to hypoxia inducible factor (HIF) 1 in clinical breast cancer samples [5]. The upregulation of SLC7A5 appears to be predominantly linked to cell survival in estrogen receptor positive (ER^+^) breast cancer subtypes, supporting amino acid metabolism during nutritional stress [6, 7]. Morotti et al. has shown increased SLC7A5 mRNA levels in breast cancer cell lines during hypoxic conditions (0.1% O_2_) [8]. Hypoxia-mimetic agents such as CoCl_2_ is a suggested in vitro model [9, 10]. For this report, we aimed to evaluate cellular responses to hypoxia-mimetic by CoCl_2_ in ER^+^ breast cancer cell lines and the possible influence on SLC7A5 expression.

## Methods

### Cell culture conditions

The ER^+^ breast cancer cell lines MCF7 (HTB-22™), T-47D (HTB-133™), BT-474 (HTB-20™) and ZR-75-1 (CRL-1500™) were obtained through the American Type Culture Collection (ATCC, Manassas, VA, U.S.A.). The cells were cultured in RPMI-1640 GlutaMAX™ medium (Gibco, Thermo Fisher Scientific, Waltham, MA, U.S.A.) supplemented with 10% fetal bovine serum (FBS), 0.01 mg/ml human recombinant insulin (Gibco) and penicillin (50 U/ml)-streptomycin (50 µg/ml) (Gibco). Cells were cultured in humidified atmosphere with 5% CO_2_ at 37 °C.

### Cell viability assay

The in vitro effect of CoCl_2_ on cell viability was evaluated using the CellTiter Blue® assay (Promega, Nacka, Sweden) according to manufacturer’s instructions. Cells were seeded onto 96-well plates (10^4^ cells/well). After 24 h, the cells were exposed to different concentrations of CoCl_2_ (Sigma-Aldrich, Saint Louis, MO, U.S.A.), dissolved in sterile Milli-Q H_2_O, varying from 0.1 to 1000 µM in triplicate for 48 h. The viability analysis was repeated once using cells from a different passage (n = 6). The effect from the CoCl_2_ was controlled against medium complemented with 0.1% sterile Milli-Q H_2_O (hereafter H_2_O). Fluorescence was measured using the Cytation™ 3 Cell Imaging Multi-Mode Reader (BioTek Instruments, Winooski, VT, U.S.A.) together with the Gen5 Software (BioTek).

### Gene expression analysis

The cells were seeded onto six-well plates with a density of 3 × 10^5^ cells per well. After 24 h the medium was changed to new medium with either 100 µM of CoCl_2_, based on viability test and previously established concentration for cell culture use [11], or 0.1% H_2_O as a vehicle control, and incubated further 48 h. The experiment was set in triplicate and was repeated once using cells from a different passage (n = 6). Total RNA was extracted using the RNeasy Plus Mini Kit (Qiagen, Hilden, Germany) according to the manufacturer’s instructions. Total RNA (500 ng) was converted to cDNA using the High Capacity cDNA Reverse Transcription Kit® (Applied Biosystems, Foster City, CA, USA) with a 2720 Thermal Cycler (Applied Biosystems). Gene expression analyses were run on duplicate sample cDNA (10 ng) with mixture of TaqMan gene expression assays, targeting SLC7A5 (Hs01001183_m1), MKI67 (Hs00606991_m1), MTOR (Hs00234508_m1) as well as the established hypoxia markers VEGF (Hs00900055_m1) and HIF1A (Hs00153153_m1) and TaqMan Fast Advanced Master Mix (Applied Biosystems). The mixtures and samples were set onto MicroAmp™ Optical 384-well plates and underwent 40 cycles of amplification using QuantStudio™ 7 Flex Real-Time PCR System (Applied Biosystems) according to manufacturer’s protocol. Extraction Non-Template Control (ENTC), Reverse Transcriptase negative (RT-) control, Non-Template Control (NTC) as well as three internal controls were run on each plate. The quantification cycle (Cq) threshold was automatically set and the expression of the target genes was normalized against a mean of the two reference genes (TOP1, Hs00243257_m1 and CYC1 Hs00357717_m1). Duplicate samples with a standard deviation (SD) value of ≥ 0.167 were re-analyzed. Fold change gene expression (CoCl_2_ vs. control) was calculated using the method 2^− ΔΔCq^ [12].

### Immunofluorescence staining

The cells were cultured in 8-well Permanox® Chamber Slides™ (Lab-Tek®, Thermo Fisher) at a density of 20 000 cells/well for 24 h and then exposed to either 100 µM of CoCl_2_ or 0.1% H_2_O for another 48 h. The Image-IT Fixation/Permeabilization Kit (ThermoFisher Scientific) was used followed by 1 h incubation with a rabbit monoclonal anti-SLC7A5 antibody diluted 1:500 (#ab208776, Abcam Cambridge, UK) and then 1 h with AffiniPure anti-rabbit Cy™3-labeled secondary antibody 1:400 (#711-165-152, Jackson ImmunoResearch Europe Ltd, Cambridge, UK). Nuclei were stained with 1 µg/mL DAPI (ThermoFisher Scientific) and F-actin with ActinGreen™ 488 ReadyProbes™ Reagent (AlexaFluor™ 488 phalloidin, Invitrogen, Carlsbad, CA, U.S.A.) for 15 min. All reactions were carried out at room temperature and the cells were washed with PBS + 0.1% Tween20 (Sigma-Aldrich, Saint Louis, MO, U.S.A.) between all steps. Confocal images were acquired using a TCS SP8 confocal microscope (Leica Microsystems GmbH, Wetzlar, Germany) and the software Leica Application Suite X version 3.4. All images were collected using identical instrument settings. The level of integrated density of SLC7A5 (mean gray value * area) was measured in raw images using the open source software Fiji (ImageJ) [13]. Briefly, five circled areas were positioned in different cell regions and three areas outside of the cells (background correction) was measured. The corrected total cell fluorescence was calculated in each region with SLC7A5 expression by subtracting the area * mean gray value of background readings from the integrated density.

### Statistical analyses

For differences in cell viability and corrected total cell fluorescence, Mann-Whitney U-test was used. The results are presented as median values and 95% confidence intervals (CI). Differences in fold change gene expression were tested using multiple t-test with the Holm-Sidak method for correction of multiple comparisons with results presented as adjusted *p*-values and graphs with mean values and standard deviations (SD). Data analysis was performed using GraphPad Prism version 7.03 (GraphPad Software, La Jolla, CA, U.S.A.). For all analyses, *p*-values < 0.05 were considered statistically significant. In figures, asterisks represent the following *p*-values; * = p < 0.05, ** = p < 0.01, *** = p < 0.001 and **** = p < 0.0001.

## Results

### Effect of CoCl_2_ on cell viability

**Fig. 1:**
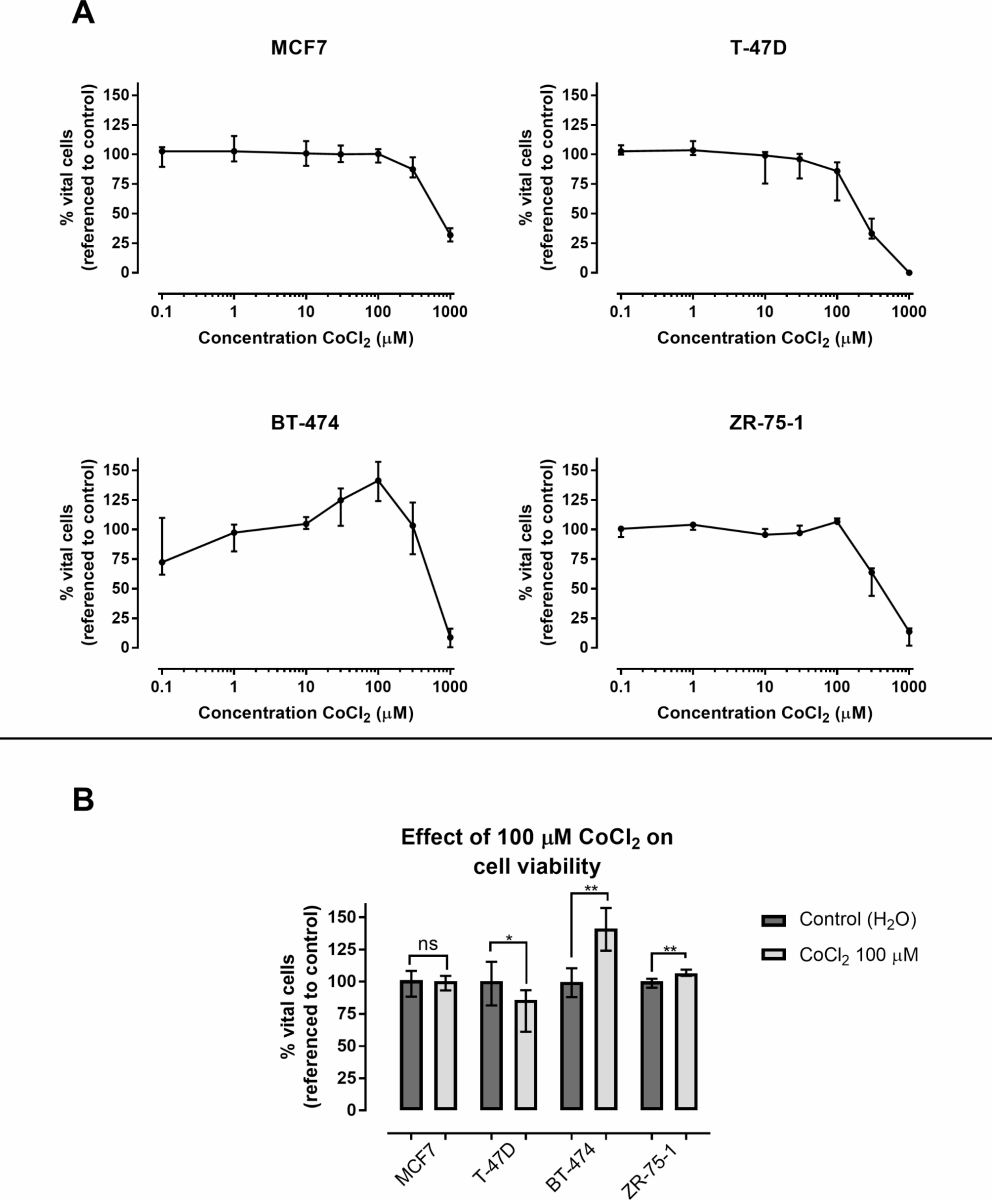
The effect of CoCl_2_ on viability. (**A**) Cell viability of MCF7, T-47D, BT-474 and ZR-75-1 cells in % after 48 h exposure to 0.1–1000 µM CoCl_2_. The bars and whiskers represent mean values with 95% confidence intervals (95% CI). The fluorescence value (i.e. viability) in each biological replicate (n = 6) was tested against the mean of the controls (n = 6). The mean of the controls was set to 100%. (**B**) Comparison of viability at 100 µM CoCl_2_ in MCF7 (p = 0.82), T-47D (p = 0.015), BT-474 (p = 0.0022) and in ZR-75-1 (p = 0.0022). The bars represent median values and the whiskers represent 95% CI. * = p < 0.05, ** = p < 0.01, ns = non-significant

Cell viability was analyzed in order to assess if CoCl_2_ introduced toxic effects (Fig. 1A). MCF7 cells exposed to 100 µM CoCl_2_ did not show any difference in viability compared to cells exposed to 0.1% H_2_O, though viability decreased to 86% in T-47D cells (p < 0.05, Fig. 1B). In BT-474 cells the cell viability increased to 141% (p < 0.01) and in ZR-75-1 cells to 107% (p < 0.01, Fig. 1B).

### Effect of hypoxia-mimetic on gene expression

**Fig. 2:**
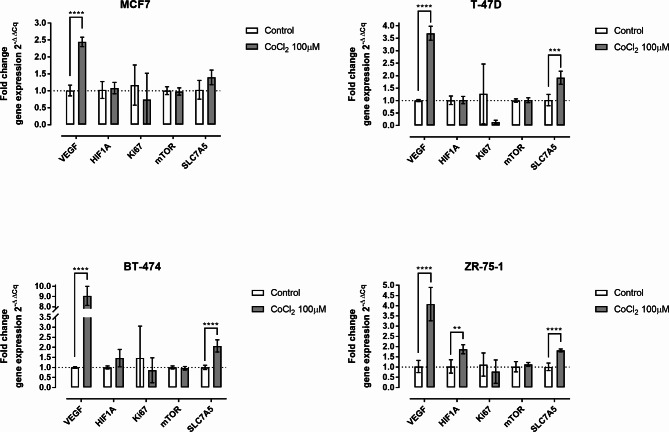
The effect of CoCl_2_ on gene expressions. The relative gene expression of VEGF, HIF1A, Ki67, mTOR and SLC7A5 in MCF7 (n = 6), T-47D (n = 6), BT-474 (n = 6) and ZR-75-1 cells (n = 6). The bars represent mean values and the whiskers represent the standard deviation. Fold change gene expression is shown on the Y-axis and adjusted *p*-values < 0.01 are represented by **, < 0.001 by *** and < 0.0001 by ****

After 48 h of exposure to CoCl_2_, the hypoxia marker VEGF showed a significant increase of at least 2-fold in all cell lines (Fig. 2), with the lowest increase observed in MCF7 cells and the highest in BT474 cells. HIF1A was found to be increased only in ZR-75-1 cells (mean FC = 1.88, SD = 0.22, p < 0.01). No statistical differences in gene expression were observed for MKI67 or mTOR, although MKI67 appeared to decrease in all cell lines, disregarding the high standard deviations. SLC7A5 expression showed a significant increase in T-47D, BT-474 and ZR-75-1. However, there was no significant change in SLC7A5 expression in MCF7 cells. Compared to control cells, SLC7A5 exhibited a mean FC of 1.92 (SD = 0.26, p < 0.001) in T47D cells, 1.82 (SD = 0.071, p < 0.0001) in ZR-75-1 cells and 2.07 (SD = 0.30, p < 0.0001) in BT-474 cells.

### Effect of hypoxia-mimetic on SLC7A5 protein expression

**Fig. 3:**
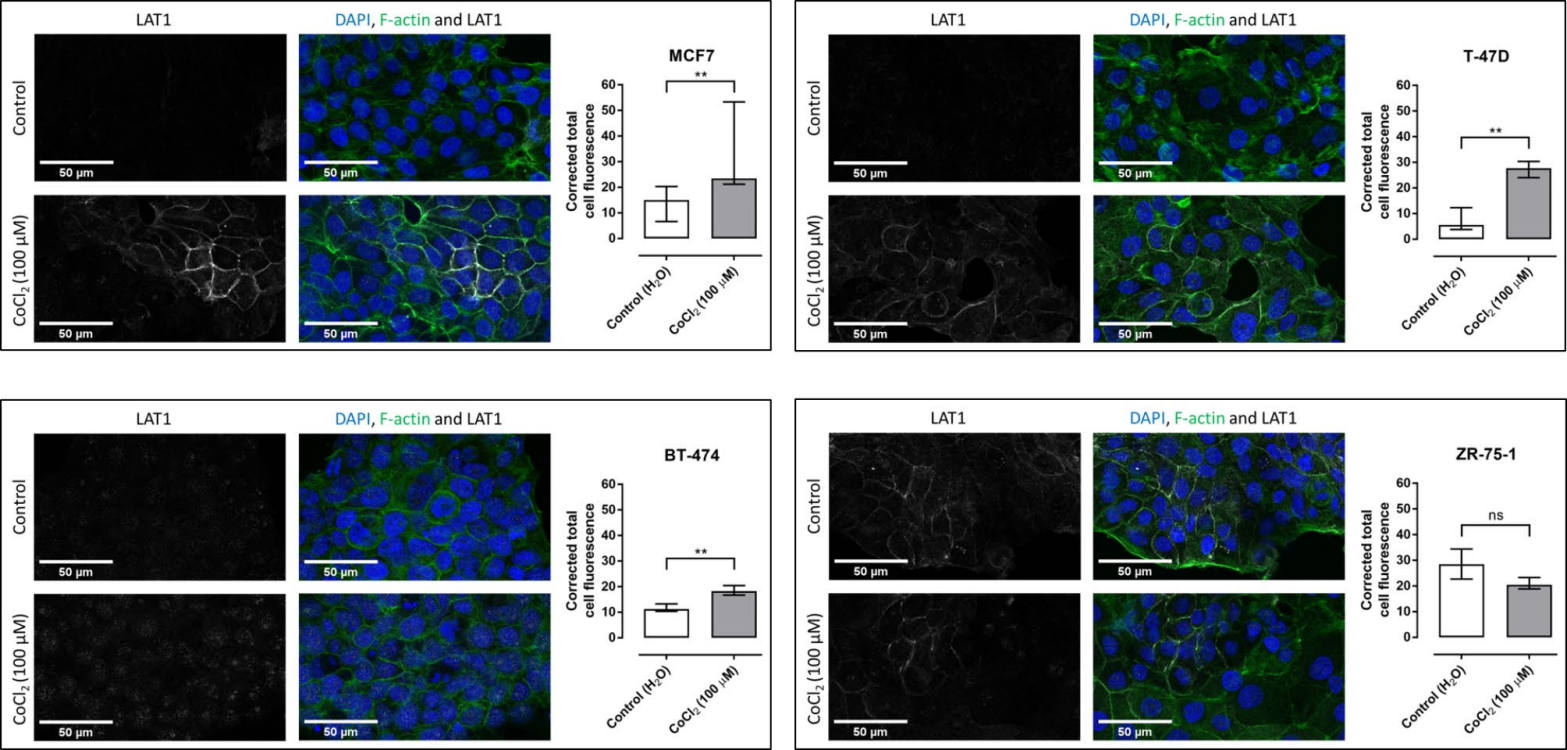
The effect of CoCl_2_ on SLC7A5 protein expression. Raw confocal images of SLC7A5 expression (gray) in MCF7 cells (upper left), T-47D cells (upper right), BT-474 cells (lower left) and ZR-75-1 cells (lower right). The white scale bars in bottom left corners of microscope images represent 50 μm. The graphs show median corrected total cell fluorescence (bars) with interquartile range (whiskers). *p*-values < 0.01 are represented by **. Ns = non-significant

The cell lines MCF7, T-47D and ZR-75-1 demonstrated higher basal expression of SLC7A5 than BT-474 (Fig. 3). Exposure to CoCl_2_ for 48 h resulted in significant increase in SLC7A5 protein expression in MCF7, T-47D and BT-474 cells. In ZR-75-1 cells, no significant difference was observed.

## Discussion

In this report, we evaluated hypoxia-mimetic by CoCl_2_ in the ER^+^ breast cancer cell lines MCF7, T-47D, BT-474 and ZR-75-1. The use of CoCl_2_ as a hypoxia model is well-established as it stabilizes HIF-1α and HIF-2α under normal oxygen levels [14]. Exposure to 100 µM CoCl_2_ resulted in increased gene expression of SLC7A5 in three out of four cell lines, with no significant change in MCF7 cells. Nevertheless, protein expression of SLC7A5 increased significantly in MCF7, T-47D and BT-474 cells, but not in ZR-75-1 cells. These findings indicate that SLC7A5 expression is regulated by hypoxic condition.

Cellular hypoxia occurs when oxygen levels range between 0.5 and 2% [15]. Morotti et al., explored endocrine resistance and the effect of hypoxia at 0.1% O_2_ in ten different breast cancer cell lines [8]. They consistently observed SLC7A5 upregulation in nine cell lines, including the four used in present report. SLC7A5 upregulation may serve as an adaptation to hypoxia, which has previously been suggested in clinical breast cancer samples [5].

Vascular endothelial growth factor (VEGF) is a recognized marker of cellular hypoxia at both translational and transcriptional level [15]. We found that all four cell lines showed significantly increased VEGF gene expression following CoCl_2_ exposure. The gene expression of HIF1A was only upregulated in ZR-75-1 cells. Decreased oxygen levels affect HIF-1α protein stability and subcellular localization rather than its gene expression, which could explain this result [16]. As VEGF and HIF-1α expression do not necessarily correlate, our findings are consistent with the existing literature and suggest that the CoCl_2_- model reliably attains a hypoxia-like condition [17, 18].

Rana et al. (2019) used CoCl_2_ as a hypoxic model in MCF7 and MDA-MB-231 cells and demonstrated that the proliferation increased, reaching its peak at concentrations of 150 µM for MCF7 and 25 µM for MDA-MB-231 [18]. Our results showed increased viability of BT-474 and ZR-75-1 at 100 µM CoCl_2_, while MCF7 and T-47D did not show the same response. All four cell lines are classified as ER^+^, with MCF7 and T-47D being HER2-negative (luminal A) and BT-474 and ZR-75-1 being HER2-positive (luminal B) [19, 20]. CoCl_2_ activates targets downstream of HER2 (PI3K/Akt) which could possibly explain the increased viability observed in the luminal B cell lines [21]. However, we were not able to show any statistical significance change in gene expression of the proliferation marker MKI67.

mTOR serves as a downstream mediator of cellular response to environmental factors such as nutrient availability and oxygen levels. It interacts with subunits to form mTOR complex 1 and 2 (mTORC1/mTORC2), with mTORC1 known for regulating cell growth, autophagy, mitochondrial respiration and amino acid metabolism [22]. Importantly, SLC7A5 imports the amino acid leucine, which promotes mTORC1 activity [23]. Although the activity of mTOR signaling typically decreases in hypoxic cells, our results did not demonstrate any changes in mTOR gene expression following CoCl_2_ exposure. In conclusion our study confirms increased SLC7A5 levels in breast cancer cells under hypoxia-mimetic conditions induced by CoCl_2_, in line with existing research. This suggests a potential of CoCl_2_ to upregulate SLC7A5 and exploring LAT1-inhibiting drug impacts on breast cancer cells, offering valuable insights for future studies.

### Limitations

There are several limitations of using CoCl_2_ as a model for hypoxia in cell culture experiments. CoCl_2_ does not replicate all mechanisms involved in the cellular properties presented at low oxygen levels. True hypoxia triggers intricate signaling pathways leading to acute and chronic effects, molecular changes and cellular adaption. While CoCl_2_ does mimic certain aspects of these responses, it falls short of reproducing the full spectrum of changes associated with true hypoxia. Also, the use of CoCl_2_ may introduce unpredictable off-target effect. There are also limitations of extrapolating results from cell culture experiments to an in vivo setting. A true hypoxic environment characterized by low oxygen levels, coupled with the presence of stromal components surrounding tumor cells, might induce changes, such as metabolic reprogramming, that cannot be adequately observed using a chemical inducer of a hypoxia-like condition.

## Data Availability

All raw data are available from the corresponding author on reasonable request.

## Abbreviations

CoCl_2_: Cobalt chloride
ER: Estrogen receptor
HER2: Human epidermal growth factor receptor 2
HIF: Hypoxia–inducible factor
LAT1: Large neutral amino acids transporter small subunit 1
MKI67: Marker of proliferation Ki–67
mTOR: Mechanistic target of rapamycin
qPCR: Quantitative polymerase chain reaction
SLC7A5: Solute carrier family 7 member 5
VEGF: Vascular endothelial growth factor
